# Cognitive impairment, depression, and anxiety in systemic sclerosis

**DOI:** 10.3389/fmed.2026.1795138

**Published:** 2026-04-27

**Authors:** Vera Szekanecz, Laura Lekli, Lilla Bokor, Zsófia Kardos, Vera Kitti Kardos, Katalin Hodosi, Szilvia Szamosi, Gabriella Szűcs, Csaba Oláh, Ágnes Horváth, Judit Molnár, Zoltán Szekanecz

**Affiliations:** 1 Department of Behavioural Sciences, Faculty of Medicine, University of Debrecen, Debrecen, Hungary; 2 Centre of Clinical Psychology, University of Debrecen Clinical Centre, Debrecen, Hungary; 3 Borsod-Abaúj-Zemplén County Central Hospital and University Teaching Hospital, Miskolc, Hungary; 4 Robert Bosch Energy and Body Systems Kft., Miskolc, Hungary; 5 Department of Rheumatology, Faculty of Medicine, University of Debrecen, Debrecen, Hungary

**Keywords:** anxiety, cognitive dysfunction, depression, psychology - study & teaching, systemic sclerosis

## Abstract

**Background:**

Systemic sclerosis (SSc) has been associated with cognitive dysfunction (CD), depression and anxiety. We conducted a comprehensive study by using multiple standardized tests in a subset of SSc patients compared with rheumatoid arthritis (RA) and healthy controls. Moreover, we correlated cognitive scores various other parameters in SSc.

**Patients and methods:**

Thirty SSc patients were recruited for the study. Data of 40 RA patients and 30 healthy individuals from our previous study were used as controls. Montreal Cognitive Assessment (MoCA), Trail Making Test (TMT), Victoria Stroop Test (VST), and Wechsler Adult Intelligence Scale version 4 (WAIS-IV) tests were used for CD; Beck Depression Inventory (BDI) for depression; and Spielberger State-Trait Anxiety Inventory (STAI) for anxiety.

**Results:**

MoCA total score and TMT-A time were similar in SSc vs. RA or controls (*p* > 0.05). TMT-B time was longer in SSc vs. controls (*p* < 0.001), but similar vs. RA (*p* = 0.343). VST-A time was also longer in SSc vs. controls (*p* = 0.003) but similar vs. RA (*p* = 0.146). Similar observations were made regarding VST-B time (SSc vs. controls, *p* < 0.001) and VST-C time (SSc vs. controls, *p* < 0.001). BDI values were similar in SSc vs. controls (*p* = 0.458) but lower vs. RA (*p* = 0.035). STAI-S (*p* = 0.011) and STAI-T (*p* = 0.006) values were significantly higher in SSc vs. controls. Several associations were identified between age, sex, alcohol intake, SSc-ILD, ANA positivity vs. various cognitive scores.

**Conclusion:**

Our findings may suggest subtle differences in specific cognitive domains, particularly executive functions, rather than evidence of global cognitive impairment in patients with SSc. SSc has also been associated with anxiety symptoms. These states may be associated with some disease-related factors. Thus, tailored cognitive behavioral therapy may be useful for our SSc patients.

## Introduction

Cognitive function includes orientation, attention and concentration, judgment and problem solving, memory, visual, verbal, as well as executive functions (1). For people with chronic conditions, such as autoimmune-inflammatory rheumatic and musculoskeletal diseases (RMDs), intact cognitive functioning is crucial for performing key daily tasks (2). Many RMDs including rheumatoid arthritis (RA), systemic lupus erythematosus (SLE) and systemic sclerosis (SSc) have been associated with cognitive dysfunction (CD), as well as depression and anxiety [reviewed in (1–4)]. While there have been relatively many studies carried out in RA (1, 2, 5–13) and SLE (2, 6, 12, 14–18) regarding CD, depression, anxiety and the use of cognitive behavioral therapies (CBT), there have been more limited number of publications in SSc in this respect (2, 19–26).

Historically the central nervous system was considered largely unaffected in SSc. In fact, emotional/mental and cognitive functioning are clearly important for SSc patients. For example, morphea is relevant in terms of functional and cosmetic burden. Emotional stress has been recognized as a factor in the development of SSc (2, 27). More specific issues occur due to the impact of SSc on physical appearance and function. SSc patients carry difficulties related to eating, working or participating in normal sociable activities. This impact has been clarified by studies using validated instruments to assess burden (2, 28). Clinical interventions aiming at improving facial appearance, such as autologous fat transfer, can significantly improve psychological wellbeing (2, 29). SSc patient often report uncertainty about the future, fear of becoming physically disabled, and a reduced life expectancy (30).

Cognitive dysfunction, as well as anxiety, depression and mood changes have been associated with the disease process in SSc (2, 24). One study found evidence of CD in SSc patients compared with matched healthy controls and that this associated with worse vascular disease (19). In addition, SSc is associated with Raynaud’s phenomenon and there have been several reports of mental or cognitive impact of cold exposure (2, 4). One group found association of CD with antinuclear antibody (ANA) positivity (24). In studies reporting CD in SSc, this condition has been associated with lower education level, malnutrition, greater fatigue, functional and work disability and worse quality of life (22, 23, 31–34). CD has been partially linked to depressive symptoms (22). In a neurosonological study, cerebrovascular pathology was associated with CD (35). CD might occur early in SSc. In one study, CD was assessed in 20 SSc patients with no known clinical neurological manifestations. The Mini-Mental State Examination (MMSE) found CD in 65% of patients. This was associated with the Medsger vascular severity vascular score. Thus, subclinical CD can occur in SSc (19). One case study evaluated the application of CBT as a part of an interdisciplinary care program also including physical and occupational therapy, as well as specialized nurse care (30). Finally, very recently, another group in Hungary published results of a detailed assessment of risk factors of CD in SSc (21). Krampek et al. (21) evaluated SSc patients with a mean disease duration of 10.5 years and healthy controls in a 1-year follow-up study. Using four tests, they found no CD in the early stages of SSc, even in severe patients with diffuse cutaneous SSc (dcSSc). The study identified risk factors that may lead to a subsequent decline in cognitive function, such as age, degree of education, pain intensity, employment status, hypertension, cardiac function and muscle strength (21). Many groups reported depression and anxiety in various SSc cohorts (25, 36–39).

To address the ongoing controversy regarding the occurrence of CD in SSc, we conducted a comprehensive study using multiple standardized tests in a subset of Hungarian patients with SSc compared to those with RA and healthy controls. We used RA as a positive disease control, based on its association with CD, depression and anxiety (1, 2, 7, 13, 40). The novelty of our study is that we used multiple tests for CD, as well as depressive and anxiety symptoms. Moreover, we correlated these results with various clinico-epidemiological parameters and laboratory biomarkers in our SSc patients.

## Patients and methods

### Patients and controls

Altogether 30 SSc patients undergoing regular follow-ups at the University of Debrecen were recruited for the study. None of the SSc patients and controls (see later) had any previous vascular events, known cerebrovascular or mental disorders. The major characteristics of these 30 SSc patients are shown in Table 1. We included 25 females and five males (5:1). The mean age of the patients was 61.5 ± 9.0 (range: 42–81) years. Their mean disease duration was 19.9 ± 8.8 (range: 4–31) years.

**TABLE 1:** Patient characteristics.

Characteristics	SSc	RA*	Control*
*n*	30	40	30
Female:male	25:5	40:0	30:0
Age (years)	61.5 ± 9.0	60.7 ± 9.5	60.9 ± 7.2
Disease duration (years)	19.9 ± 8.8	11.6 ± 7.6	-
Years at school (years)	12.1 ± 4.1	11.4 ± 3.0	12.0 ± 1.6
Smoker (%)	3.4	16.6	8.2
Alcohol consumption (%)	26.7	12.5	29.7
dcSSc:lcSSc	7:23	–	–
ILD/fibrosis, *n* (%)	23 (77)	N/A	N/A
PAH, *n* (%)	2 (7)	N/A	N/A
Digital ulcer, *n* (%)	14 (47)	N/A	N/A
ANA positivity, *n* (%)	23 (77)	N/A	N/A
Anti-Scl70 positivity, *n* (%)	15 (50)	N/A	N/A
Anti-centromere positivity, *n* (%)	2 (7)	N/A	N/A

*Data taken from Oláh et al., (Rheumatol Int, 2019). ANA, antinuclear antibody; dc, diffuse cutaneous; ILD, interstitial lung disease; lc, limited cutaneous; PAH, pulmonary arterial hypertension; RA, rheumatoid arthritis; SSc, systemic sclerosis.

For this study, 40 RA patients and 30 healthy individuals were chosen as historical positive and negative controls from our previous study, respectively (13). Because those data were already published, we only used the data for statistical comparison with SSc patients. The age of RA patients (60.7 ± 9.5 years) and healthy controls (60.9 ± 7.2 years) was not significantly different from that of SSc patients (Table 1). Moreover, the testing conditions, including the clinical setting and the expertise of the examiners, were identical for the SSc patients in the present study and in the previous study. The very same inclusion and exclusion criteria were used (13). Specifically, the inclusion criteria were: age ≥ 18 years; definitive diagnosis of SSc or RA; stable dose of all DMARDs; informed consent. The exclusion criteria included pregnancy and/or breast feeding; any previous vascular events including cerebrovascular diseases, as well as mental disorders; history of skull trauma; known depression and other mood disorders; claustrophobia. The patients did not receive any medications that influence the central nervous system, such as corticosteroids or antidepressants.

Ethical approval (No. 1046-63/2015) was obtained from the Regional/Institutional Review Board of University of Miskolc. All patients and controls signed informed consent. The study was performed according to the Declaration of Helsinki.

### Tests of cognitive function, depression, and anxiety

The following standardized tests also translated to Hungarian were used (41):

The Montreal Cognitive Assesment (MoCA) test is suitable for the determination of cognitive abilities in general, such as memory, spatial-visual abilities, attention, concentration, language ability, executive functions. The normal value is ≥26 and higher values indicate better cognitive function. A score below 26 can is indicative for mild cognitive impairment and early-onset dementia (41, 42).

Trail Making Test (TMT) measures the visual attention, speed of visual processing, mental flexibility, and executive functions. In part A of the TMT (TMT-A) assessing mainly attention (visuomotor processing speed and psychomotor skills) numbers are presented on a page in random array. The patient is instructed to connect the numbers in ascending order as quickly as possible. Part B (TMT-B) determines executive functions such as cognitive flexibility and response inhibition. Here both numbers and letters are on the sheet, and the examinee must alternate connecting numbers and letters in sequence. The score on each part is the time in seconds (sec) required to complete the task. Higher number of numbers and letters identified, and shorter time indicates better function (41, 43).

The Victoria Stroop Test (VST) is a brief version of the Stroop task. The Stroop task measures executive functions by testing cognitive flexibility, selective attention and response inhibition. The Victoria Stroop Test (VST) contains 24 items in each of the following conditions: naming the color of dots (VST-A), of neutral words (VST-B), and of color words printed in contrasting colors (VST-C). We determined the time in sec (41, 43).

The Wechsler Adult Intelligence Scale version 4 (WAIS-IV) provides information on intelligence and cognitive ability. The test consists of 10 main subtests and five supplemental subtests. We used the Digit Symbols subtest of the Hungarian version, assessing information processing speed, attention, and visual-motor coordination. The number of correct symbols reproduced within the time-limit is measured. Numbers are then converted to Q points (age-adjusted standard scores) (44–46).

The Beck Depression Inventory (BDI) is a self-report 21-item rating inventory that measures characteristic attitudes and symptoms of depression. Higher BDI values represent higher degree of depression (47, 48).

The Spielberger State-Trait Anxiety Inventory (STAI) is a commonly used self-scored measure of trait and state anxiety. STAI-S indicates state (current status) and STAI-T measures trait (general status) anxiety. Lower STAI-S or STAI-T values represent less trait and state anxiety (49, 50).

All tests were performed by our clinical psychologists (VS, LL, LB).

### Clinical and laboratory assessments

A detailed history taking and physical examination of each SSc patient was performed. Information was obtained on age, sex, diffuse (dcSSC) or limited cutaneous SSc (lcSSc) type, and disease duration. We inquired about regular smoking and alcohol consumption during the past 2 years. We also calculated the number of years in education (Table 1). Lung involvement including interstitial lung disease (ILD), pulmonary fibrosis (PF) or pulmonary arterial hypertension (PAH), cardiovascular, renal involvement, and digital ulcers (DU) were determined according to the standard protocol using clinical and laboratory measures, high resolution computed tomography (HRCT), lung function tests (LFT) and echocardiography. Antinuclear (ANA), anti-centromere (ACA) and anti-topoisomerase I (anti-Scl70) antibodies were determined in the Department of Laboratory Medicine, University of Debrecen using standard methods.

### Statistical analysis

The statistical analysis was processed with IBM SPSS 22 software. The data are expressed as the mean ± SD and frequencies and percentages. Continuous variables were compared by Mann-Whitney test. Simple correlations were determined by Spearman’s analysis. Multiple linear regression analysis using the stepwise method was used to determine correlations and independent associations between parameters. Cognitive test results were the dependent variables and several other clinical, laboratory and imaging parameters were independent variables. The β standardized linear coefficients showing linear correlations between two parameters were determined. The B (+95% CI) regression coefficient indicated independent association between the dependent and independent variable during changes. Power calculation using the G-Power software showed that in the SSc-control comparison the power was 52%–97% for *n* = 30–28. Regarding correlations, the calculated power was 71%–91% at *n* = 30. We also performed Bonferroni correction of the results. The resulting *p*-value is 0.008 for statistical significance.

## Results

### Basic characteristics of SSc patients and controls

The demographic, clinical and immunological characteristics of SSc patients are presented in Table 1. As described above, the ratio of females and males was 5:1. There was no significant difference between SSc vs. RA vs. controls in age (*p* > 0.05). SSc patients had somewhat longer disease duration compared to RA patients (*p* < 0.05) (Table 1).

Regarding patient history, we calculated years spent in education. There was no significant difference between SSc patients (12.1 ± 4.1 years), RA patients (11.4 ± 3.0 years) and controls (12.0 ± 1.6 years) (*p* > 0.05) (Table 1). Only 3.4% of SSc patients were current smokers, compared with 16.6% of RA patients (*p* < 0.05) and 8.2% of controls (*p* < 0.05). In contrast, more SSc patients (26.7%) and controls (29.7%) consumed some, but not much alcohol compared to RA patients (12.5%; *p* < 0.05) (Table 1).

With respect to disease subtype, 7 SSc patients had dcSSc (23.3%) and 23 had lcSSc (76.7%). Altogether 77% of SSc patients had ILD/PF, 7% had PAH and 47% had DU. Only five patients (17%) had renal involvement, but not scleroderma renal crisis. Regarding immunolaboratory, 77% of SSc patients were ANA positive, 50% anti-Scl70 positive and 7% ACA positive (Table 1).

### Results of cognitive function, depression, and anxiety tests

**FIGURE 1:**
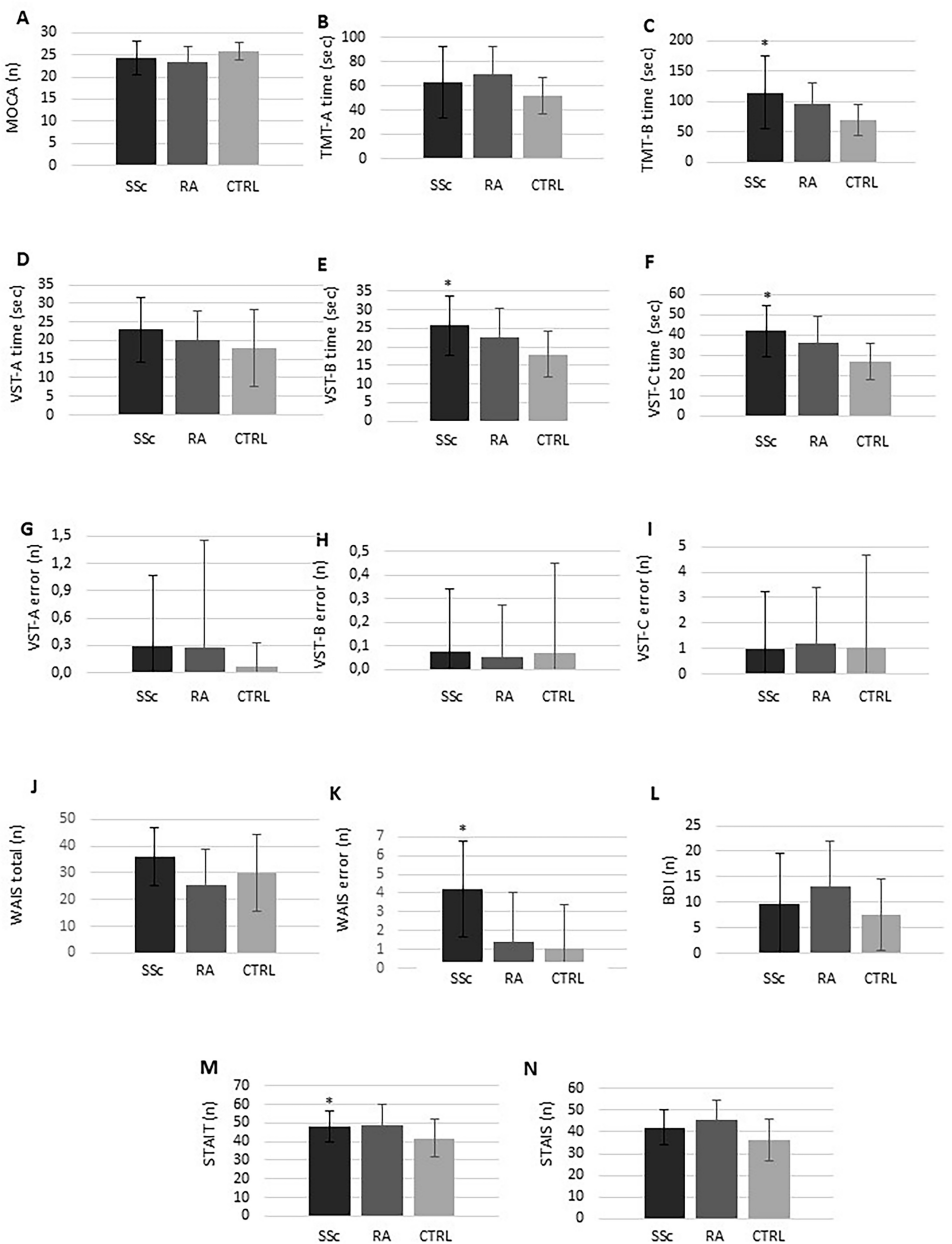
Results of cognitive function **(A–K)**, depression **(L)**, and anxiety **(M,N)** tests in systemic sclerosis (SSc), rheumatoid arthritis (RA) and healthy controls. RA and healthy control historical data were obtained from Olah et al. (13) and only used for comparative purposes. *p < 0.05.

Among cognitive function tests, the MoCA total score was similar in SSc patients (24.3 ± 3.8) as in RA patients (23.2 ± 3.6; *p* = 0.208) or controls (25.9 ± 2.0; *p* = 0.178; Figure 1A). The cutoff value of MoCA is 26, values ≥ 26 indicating normal status. In SSc the mean MoCA was 24.3 ± 3.8, in general, somewhat impaired. Abnormally low (<26) MoCA was observed in 53% of SSc patients. With respect to TMT, the time of identification in the TMT-A part was similar in SSc (62.9 ± 29.1 s) as in RA (69.1 ± 23.0 s; *p* = 0.317) and controls (51.6 ± 14.8 s; *p* = 0.113; Figure 1B). In the TMT-B part, time of identification was significantly longer (abnormal) in SSc patients (114.2 ± 59.8 s) versus controls (68.4 ± 25.4 s; *p* = 0.001) but similar versus RA (95.8 ± 35.4 s; *p* = 0.343; Figure 1C). Regarding VST, in VST-A, SSc patients (22.9 ± 8.8 s) needed more time (abnormal) to complete the task than controls (18.0 ± 10.4 s; *p* = 0.003) but similar length of time as RA patients (20.0 ± 7.8 s; *p* = 0.146; Figure 1D). Similar observations were made regarding VST-B time (SSc [25.7 ± 8.1 s] versus controls [18.0 ± 6.0 s]: *p* < 0.001; versus RA [22.6 ± 7.7 s]: *p* = 0.111; Figure 1E) and VST-C time (SSc [41.8 ± 12.4 s] versus controls [26.5 ± 9.1 s]: *p* < 0.001; versus RA [36.0 ± 12.9]: *p* = 0.052; Figure 1F). There were no significant differences between SSc, RA patients and controls in VST-A errors (SSc: 0.30 ± 0.78, RA: 0.28 ± 1.17, controls: 0.07 ± 0.26; *p* > 0.05; Figure 1G), VST-B errors (SSc: 0.07 ± 0.27, RA: 0.05 ± 0.22, controls: 0.07 ± 0.38; *p* > 0.05; Figure 1H), VST-C errors (SSc: 0.96 ± 2.28, RA: 1.18 ± 2.20, controls: 1.04 ± 3.27; *p* > 0.05; Figure 1I), and WAIS total scores (SSc: 36.1 ± 10.7, RA: 25.4 ± 13.3, controls: 30.0 ± 14.3; *p* > 0.05; Figure 1J). Regarding WAIS errors, SSc patients made significantly more errors (4.23 ± 2.53) compared to controls (1.04 ± 2.32; *p* = 0.006; Figure 1K). There was no significant difference in WAIS errors in SSc compared to RA (1.44 ± 2.57; *p* = 0.046; Figure 1K).

Beck Depression Inventory values indicating the level of depression were similar in SSc patients (9.63 ± 9.94) and controls (7.68 ± 7.00; *p* = 0.458; Figure 1L) or RA patients (13.18 ± 8.55; *p* = 0.035; Figure 1L).

Finally, STAI measures state (STAI-S) and trait anxiety (STAI-T). STAI-S was somewhat, but not significantly higher in SSc (42.1 ± 8.3) versus controls (36.4 ± 9.5; *p* = 0.011; Figure 1M). On the other hand, STAI-T was significantly higher (abnormal) in SSc (48.3 ± 8.4) compared to controls (41.7 ± 10.1; *p* = 0.006; Figure 1N). There were no significant differences in STAI-S (RA: 45.8 ± 8.9; *p* = 0.164; Figure 1M) and STAI-T (RA: 48.6 ± 11.6; *p* = 0.918; Figure 1N) between SSc and RA patients.

### Correlations among various cognitive function, depressive, and anxiety symptoms in SSc patients

Results of the various cognitive functions, depression and anxiety tests described above were correlated with each other within the SSc population (Table 2). As many of the cognitive function tests assess somewhat overlapping domains, numerous correlations could be identified. All correlations are seen in Table 2, we do not present them one by one here. In general, several cognitive function tests correlated with each other but, interestingly, none of them correlated with depression and anxiety tests (Table 2). Moreover, depression and anxiety tests correlated with each other, but not with cognitive function tests (Table 2).

**TABLE 2:** Significant correlations between results of cognitive function, depression, and anxiety tests (Spearman’s test).

Psychologic tests	MOCA	TMT-A time	TMT-B time	VST-A time	VST-A error	VST-B time	VST-B error	VST-C time	VST-C error	WAIS total	WAIS error	BDI	STAIT	STAIS
MOCA	–	NS	**–**	NS	NS	NS	NS	–	NS	**–**	NS	NS	NS	NS
TMT-A time	NS	–	*R* = 0.607 *p* < 0.001	*R* = 0.530 *p* = 0.004	NS	*R* = 0.497 *p* = 0.008	NS	NS	NS	*R* = −0.497 *p* = 0.006	NS	NS	NS	NS
TMT-B time	NS	*R* = 0.607 *p* < 0.001	–	*R* = 0.536 *p* = 0.004	NS	*R* = 0.521 *p* = 0.005	NS	NS	NS	*R* = −0.706 *p* < 0.001	NS	NS	NS	NS
VST-A time	NS	*R* = 0.530 *p* = 0.004	*R* = 0.536 *p* = 0.004	–	NS	*R* = 0.666 *p* < 0.001	NS	*R* = 0.771 *p* < 0.001	NS	NS	NS	NS	NS	NS
VST-A error	NS	NS	NS	NS	–	NS	NS	NS	NS	*R* = −0.632 *p* < 0.001	NS	NS	NS	NS
VST-B time	NS	*R* = 0.497 *p* = 0.008	*R* = 0.521 *p* = 0.005	*R* = 0.666 *p* < 0.001	NS	–	NS	NS	NS	NS	NS	NS	NS	NS
VST-B error	NS	NS	NS	NS	NS	NS	–	NS	NS	NS	NS	NS	NS	NS
VST-C time	NS	NS	NS	*R* = 0.771 *p* < 0.001	NS	NS	NS	–	NS	NS	NS	NS	NS	NS
VST-C error	NS	NS	NS	NS	NS	NS	NS	NS	–	NS	NS	NS	NS	NS
WAIS total	NS	*R* = −0.497 *p* = 0.006	*R* = −0.706 *p* < 0.001	NS	*R* = −0.632 *p* < 0.001	NS	NS	NS	NS	–	NS	NS	NS	NS
WAIS error	NS	NS	NS	NS	NS	NS	NS	NS	NS	NS	–	NS	NS	NS
BDI	NS	NS	NS	NS	NS	NS	NS	NS	NS	NS	NS	–	*R* = 0.565 *p* = 0.001	NS
STAIT	NS	NS	NS	NS	NS	NS	NS	NS	NS	NS	NS	*R* = 0.565 *p* = 0.001	–	*R* = 0.554 *p* = 0.002
STAIS	NS	NS	NS	NS	NS	NS	NS	NS	NS	NS	NS	NS	*R* = 0.554 *p* = 0.002	–

BDI, Beck Depression Inventory; MOCA, Montreal Cognitive Assessment; NS, non-significant; STAIT/S, State-Trait Anxiety Inventory; TMT, Trail Making Test; VST, Victoria Stroop Test; WAIS, Wechsler Adult Intelligence Scale.

### Correlations between cognitive function, depression, and anxiety test results and other demographic, clinical and laboratory parameters in SSc patients

Univariable and multivariable regression analysis were performed to determine associations between cognitive function, depression, or anxiety test scores (dependent variables) and other demographic, clinical and laboratory parameters (independent variables) within the SSc cohort (Table 3).

**TABLE 3:** Uni- and multivariable analyses of significant associations between mental tests and other parameters.

Dependent variable	Independent variable	Univariable analysis				Multivariable analysis			
		β	*P*	B	CI 95%	β	*P*	B	CI 95%
TMT-A time	Age	0.593	0.001	1.780	0.826–2.734	0.514	0.001	1.542	0.681–2.404
	Alcohol intake	0.507	0.005	23.801	7.832–39.770	0.407	0.007	38.228	11.273–65.184
TMT-B time	Male sex	0.477	0.008	82.596	23.729–141.463	0.477	0.008	82.596	23.729–141.463
VST-A time	Age	0.528	0.005	0.468	0.158–0.777	–			
WAIS total	Age	−0.697	<0.001	−0.785	−1.098 to −0.473				

TMT, Trail Making Test; VST, Victoria Stroop Test; WAIS, Wechsler Adult Intelligence Scale.

In the univariable analysis, age correlated with abnormalities of multiple cognitive test results (TMT-A, VST-A B time, and WAIS total; *p* ≤ 0.008) (Table 3). Male sex and alcohol intake also correlated with TMT-B and TMT-A time, respectively (*p* ≤ 0.008; Table 3).

Among associations identified in the univariable analysis, multivariable regression also confirmed that increasing age was linked to longer TMT-A time (*p* = 0.001; Table 3). Similarly, the correlations between male sex and TMT-B time (*p* = 0.008), and alcohol intake and TMT-A time (*p* = 0.007) remained significant in the multivariable model (Table 3).

We did not find any correlations between results of depression (BDI) and anxiety tests (STAI) and any demographic, clinical or laboratory parameters described above.

## Discussion

In this study, we assessed cognitive function, as well as SSc-related depressive and anxiety symptoms using numerous tests in a cohort of SSc patients compared to our historical RA and control cohort. Several cognitive function tests (TMT-B, VST-B, and VST-C time, as well as WAIS error), as well as STAI-T showed CD and anxiety in SSc patients compared to controls, respectively.

We performed this study mainly as there has been some controversy regarding CD in SSc patients. In general, CD, depression and anxiety have been described in association with the disease process in SSc (2, 24). Khedr et al. (19) reported impaired cognitive function in SSc patients versus healthy controls. Wuriliga et al. (24) found mild CD in SSc. Some other groups also published data on variable degree of CD in patients with SSc (19, 22, 23, 31–35). In many studies, CD was more prevalent in advanced SSc, however some reports suggest that CD may also occur in early SSc (19, 22, 23, 31–35). Furthermore, in the study of Khedr et al. (19), the MMSE test detected CD in two-third of SSc patients with no clinically overt neuropsychological disease. Thus, even subclinical CD can be associated with SSc (19). In contrast to all these studies, very recently Krampek et al. (21) have not found any signs of CD using four tests even in more advanced, severe SSc. Using multiple cognitive function tests, we confirmed the presence of CD in patients with SSc. Moreover, the mean disease duration in our cohort (almost 20 years) was longer that reported by Krampek et al. (10.5 years) (21). Finally, discrepancies might stem from the different sensitivity levels of the screening tools used in other studies (21) versus the comprehensive battery applied in the present study.

We also confirmed the presence of anxiety symptoms in our SSc patients. Scores on STAI-T scales indicated anxiety in SSc, similarly to RA, and significantly higher than in healthy controls. In contrast, depression in SSc patients were similarly low to those in healthy individuals. Thus, these findings suggest that while anxiety symptoms were evident in SSc, depressive signs were not prominent in our cohort. Alexopoulos et al. (22) reported both CD and depression in SSc. However, that group used different cognitive function and depression tests from what we did. Numerous groups reported anxiety and mood disorders associated with SSc (25, 36–39). Notably, we found no elevation in depressive symptoms among our SSc patients relative to healthy controls, despite the presence of significant anxiety and cognitive dysfunction. The results of the BDI and STAI should be interpreted as reflecting levels of depressive and anxiety symptoms rather than clinical diagnoses, and scores may also be influenced by somatic symptoms related to SSc. This pattern indicates a dissociation between these psychological domains in our cohort and suggests our SSc population may be less prone to depression than those described in some other reports.

We performed a simple correlation analysis to find associations between CD, as well as depressive and anxiety symptoms in our SSc patients. As the mechanisms of CD largely overlap, not surprisingly, numerous cognitive function tests correlated with each other. Moreover, depression and anxiety tests also showed correlations with each other. Interestingly though, in this analysis, CD did not correlate with levels of depression and anxiety. In the literature, most studies evaluated cognitive function independently from anxiety and mood disorders (4, 19–21, 23, 24, 31–35, 37), while anxiety and depression were usually studied together (25, 36–38). Alexopoulos et al. (22) linked CD to depression. This group assessed mood by the GDS depression scale and found that CD partially associated with depressive symptoms (22). Given that only one recent study has linked CD to depression, our data suggest that CD and depressive/anxiety symptoms might occur separately. There is a possibility that they might be driven by different pathological pathways in SSc.

To characterize the contribution of demographic and clinical factors, as well as laboratory biomarkers to CD, we performed uni- and multivariable regression analysis. In our study, various indicators of CD were correlated with age, male sex, and alcohol intake. We did not find any associations between depressive/anxiety symptoms and any of the studied markers. Age and alcohol intake are known denominators of cognitive decline in various diseases, as well as in the general population (1, 2, 13, 21). Krampek et al. (21) also correlated cognitive function with age in their SSc cohort. Other studies suggested the link of CD to lower education level, pain, functional impairment, work disability and worse quality of life (22, 23, 31–34).

Our study may have significant strengths and limitations. Our study provides a comprehensive assessment of neuropsychiatric symptoms in SSc, which utilized multiple, well-validated instruments to measure cognitive function, depression and anxiety tests. Unlike previous studies that evaluated CD separately from depression/anxiety, we performed the analysis of the three features concurrently. In addition, these test results were evaluated in the context of demographic, clinical and laboratory variables. The limitations of our study include the relatively low number of SSc patients, which may have influenced the statistical power. We had to use historical control groups, which approach could introduce potential biases related to differences in recruitment procedures, testing conditions, or clinical context. Lifestyle-related factors that may influence cognitive function, such as smoking and alcohol were only roughly evaluated in our SSc cohort. Being a cross-sectional analysis, the effect of treatments on cognitive function could not be prospectively assessed. Finally, while most variables could be numerically determined, alcohol intake and smoking habits were only roughly estimated.

In conclusion, our findings may suggest subtle differences in specific cognitive domains, particularly executive functions, rather than evidence of global cognitive impairment in patients with SSc. SSc has also been associated with anxiety, but less depressive symptoms as determined by multiple tests. CD and anxiety/depressive symptoms might be independent from each other. Various domains of CD dysfunction maybe associated with age, male sex, and alcohol intake. The early diagnosis of CD and anxiety is important for our SSc patients.

## Data Availability

The raw data supporting the conclusions of this article will be made available by the authors, without undue reservation.
